# Administration of Danhong Injection to diabetic *db/db* mice inhibits the development of diabetic retinopathy and nephropathy

**DOI:** 10.1038/srep11219

**Published:** 2015-06-10

**Authors:** Mengyang Liu, Quan Pan, Yuanli Chen, Xiaoxiao Yang, Buchang Zhao, Lifu Jia, Yan Zhu, Boli Zhang, Xiumei Gao, Xiaoju Li, Jihong Han, Yajun Duan

**Affiliations:** 1 State Key Laboratory of Medicinal Chemical Biology, Nankai University, Tianjin 300071, China; 2 College of Life Sciences, Nankai University, Tianjin 300071, China; 3 Collaborative Innovation Center for Biotherapy, Nankai University, Tianjin 300071, China; 4 Buchang Pharmaceutical Co. Ltd., Xi’an 712000, China; 5 Tianjin University of Traditional Chinese Medicine, Tianjin 300193, China

## Abstract

Danhong Injection (DHI), a Chinese medicine for treatment of patients with coronary heart disease, inhibits primary abdominal aortic aneurysms in apoE deficient (apoE^**−/−**^) mice. Formation of microaneurysms plays an important role in the development of diabetic retinopathy and nephropathy. It remains unknown if DHI can reduce these diabetic complications. In this study, diabetic *db/db* mice in two groups were injected with saline and DHI, respectively, for 14 weeks. Blood and tissue samples were collected to determine serum glucose, lipids and tissue structure. DHI reduced diabetes-induced body weight gain, serum cholesterol and glucose levels. In retinas, DHI blocked the shrink of whole retina and retinal sub-layers by inhibiting expression of caspase 3, matrix metalloproteinase 2 (MMP-2) and MMP-9, accumulation of carbohydrate macromolecules and formation of acellular capillaries. DHI improved renal functions by inhibiting mesangial matrix expansion, expression of vascular endothelial growth factor A, fibronectin and advanced glycation end products in kidneys. Mechanistically, DHI induced expression of glucokinase, AMPKα/phosphorylated AMPKα, insulin receptor substrate 1, fibroblast growth factor 21 and peroxisome proliferator-activated γ. Expression of genes responsible for energy expenditure was also activated by DHI. Therefore, DHI inhibits diabetic retinopathy and nephropathy by ameliorating glucose metabolism and demonstrates a potential application in clinics.

The number of diabetic patients is expected to be more than 500 million in 2030 globally^1^. Both diabetes-induced macrovascular (coronary artery disease, peripheral artery disease and stroke) and microvascular (retinopathy, nephropathy and neuropathy) complications are the major causes of morbidity and mortality of the patients^2^. The risk of both microvascular and macrovascular complications is strongly correlated to hyperglycemia, which indicates the importance of glucose homeostasis. Correspondingly, the lowering blood glucose treatment substantially reduces the risk of development of retinopathy and nephropathy^3^.

Liver plays an essential role in whole body glucose homeostasis which can be regulated by several molecules in the liver^4^. Phosphoenolpyruvate carboxykinase 1 (PCK1) and glucose-6-phosphatase (G6Pase) are the rate-limiting enzymes for the first and last step of gluconeogenesis, respectively. Overexpression of G6Pase can cause hyperglycemia and glucose intolerance in the animal model^5^. Glucokinase (GCK), the rate-limiting enzyme for glycolysis, enhances the glucose flux in the liver. Activation of AMP-activated protein kinase (AMPK) increases lipid and glucose catabolism^6^. Insulin receptor substrate 1 (IRS1) and fibroblast growth factor 21 (FGF21) are another two important molecules which can influence insulin signaling and glucose/lipid homeostasis^7^. For instance, administration of FGF21 reduces hypertriglyceridemia induced by genetic or diet-induced obesity in mice^8^,^9^. Hypertriglyceridemia, characterized by increased triglyceride (TG)-rich lipoproteins (TRLs), such as low-density lipoprotein (LDL) and very low-density lipoprotein (VLDL), is associated with different stages of diabetes^10^. It also contributes to the increased macrovascular disease, such as atherosclerosis. Therefore, lowering LDL and VLDL levels have demonstrated effectiveness in the primary and secondary prevention of diabetic vascular complications^11^.

Formation of advanced glycation end products (AGEs) is another risk factor for the development of diabetic complications^12^,^13^. Treatment with AGEs inhibitors prevents corneal structural abnormalities in diabetic rats^14^. Vascular endothelial growth factor A (VEGFA) is a major regulator of angiogenesis^15^. Associated with the development of diabetic nephropathy, the increased VEGFA expression, expanded glomerular mesangium and an excessive accumulation of extracellular matrix (ECM) proteins occur^16^.

Both the herbal Radix Salviae Miltiorrhizae (*Salvia miltiorrhiza* Bge, Labiatae, Danshen in Chinese) and Flos Carthami (*Carthamus tinctorius* L., Compositae, Honghua in Chinese) are the well-known traditional Chinese medicines. They have been used widely and successfully to treat patients with cardiovascular diseases for a long history in China^17^,^18^. Currently, both are officially listed in the Chinese Pharmacopeia^19^. Danhong Injection (DHI) is a combination product of Radix Salviae Miltiorrhizae and Flos Carthami with main components of tanshinone, tanshinol acid and safflor yellow. DHI has been industrialized, patented, and well characterized by a dual-standard quality assessment. DHI was certificated by the Sino Food and Drug Administration (SFDA) with the Country Medicine Accurate Character Z20026866^20^. We previously reported that DHI inhibited the development of aortic atherosclerotic lesions and primary abdominal aortic aneurysms in apoE deficient (apoE^−/−^) mice which further suggests the protective effects of DHI in the vascular system^21^,^22^. Both diabetic retinopathy and nephropathy are microvascular diseases. Therefore, in this study, we initially investigated if administration of DHI can inhibit the development of diabetic retinopathy and nephropathy in a type 2 diabetic animal model, *db/db* mice. We then determined the involved mechanisms, in particular the effects of DHI on signaling pathways for insulin sensitivity, metabolism of glucose, lipid and energy.

## Results

### DHI inhibits the development of diabetic retinopathy

**Figure 1:**
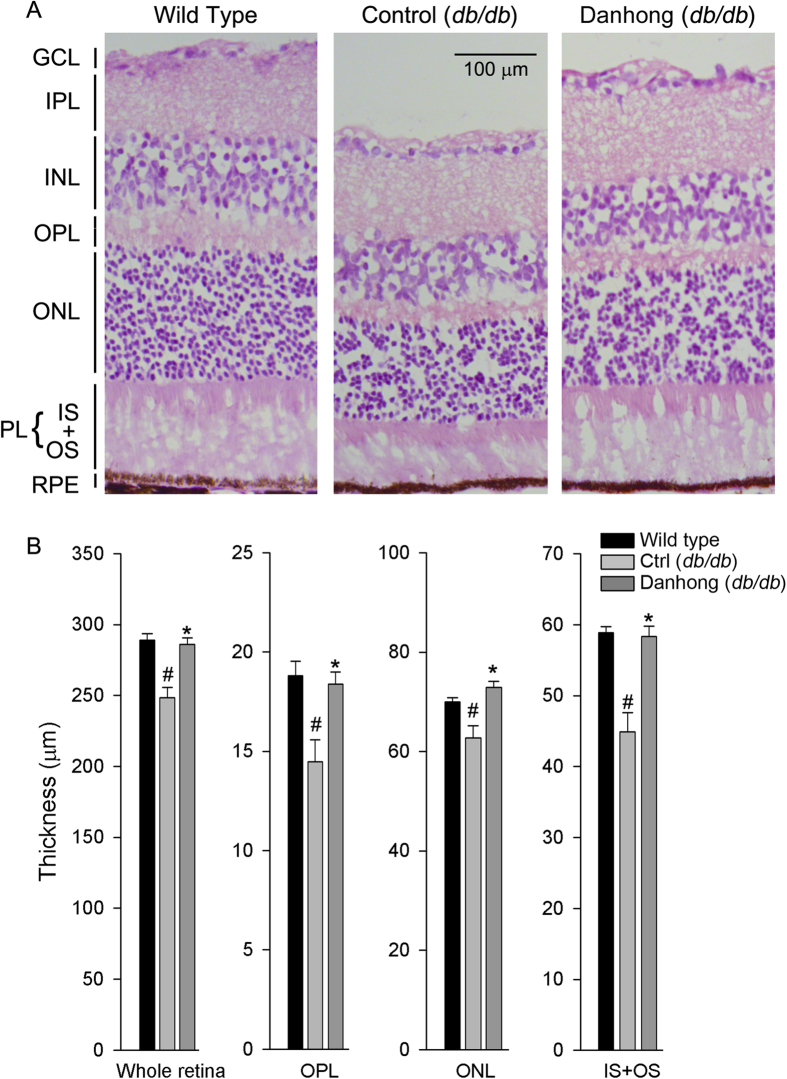
DHI ameliorates retinal abnormalities in ***db/db*** mice. Male *db/db* mice in two groups (10/group) were daily i.p. injected with saline [Control (*db/db*), 5 ml/kg body weight] or DHI [Danhong (*db/db*), 5 ml/kg body weight] for 14 weeks. Wild type mice receiving saline injection were used as a normal control. The frozen cross sections were prepared from mouse eyeballs and conducted HE staining. **A**: the representative images of retinal structure of each group; **B**: the quantitative determination of thickness of whole retina, outer plexiform layer (OPL), outer nuclear layer (ONL) and photoreceptor layer (IS + OS). #: *vs.* wild type mice; *: *vs.* control *db/db* mice, p < 0.05 (n ≥ 5).

To determine the effect of DHI on diabetic retinopathy, *db/db* mice (~6-week-old) were i.p. injected with equal volume of saline or DHI based on body weight. C57BLKS/J wild type mice at the same age received saline injection as a normal (nondiabetic) control. The treatment was continued for 14 weeks. After treatment, retina samples from all the mice were collected and prepared retinal sections followed by HE staining. The retinal thickness and structural alterations were determined with the light microscopy. The whole retinal thickness is defined as the distance from the edge of ganglion cell layer (GCL) to the edge of retinal pigment epithelium (RPE). Compared to wild type mice, Fig. 1A shows that the whole retinal thickness in control *db/db* mice was significantly reduced. However, treatment of *db/db* mice with DHI restored it to normal level indicting that DHI can maintain the integrity of retinal structure. In fact, the reduced thickness of sub-layers, such as outer plexiform layer (OPL), outer nuclear layer (ONL) and photoreceptor layer (IS + OS), in the retinas of *db/db* mice were restored to the levels of wild type mice, respectively (Fig. 1B).

**Figure 2:**
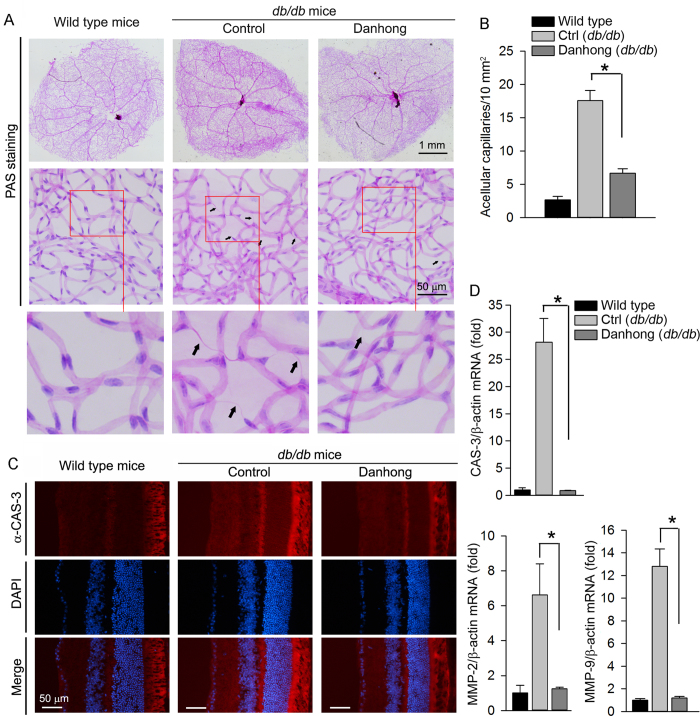
DHI inhibits formation of acellular capillaries by inhibiting CAS-3, MMP-2 and MMP-9 expression in the retinas of ***db/db*** mice. **A**: the retinal vascular network was stained with PAS and photographed; **B**: acellular capillaries indicated by black arrows in Fig. 1A were quantified. *: p < 0.05 (n = 5); **C**: CAS-3 protein expression was determined by immunofluorescent staining frozen sections of mouse retinas; **D**: total cellular RNA was extracted from retina and determined CAS-3, MMP-2 and MMP-9 mRNA expression using real time RT-PCR. *: p < 0.05 (n = 3).

Formation of acellular capillaries in the diabetic retinas was determined by PAS staining the retinal vasculature. Compared to wild type mice, acellular capillaries were increased in control *db/db* mice. However, few of acellular capillaries were found in *db/db* mice receiving DHI treatment (Fig. 2A,B).

Cellular apoptosis is a hallmark of early diabetic retinopathy in humans and animal models. High glucose levels can induce caspase 3 (CAS-3) expression which can enhance cellular apoptosis^23^. In consistent with the diabetes-mediated alterations of retinal structure (Fig. 1A), the results of immunofluorescent staining in Fig. 2C show increased CAS-3 expression in these layers with greatest in photoreceptor layer in control *db/db* mice. However, this increase was inhibited by DHI indicating the diabetes-induced apoptosis might be blocked by DHI. Activation of matrix metalloproteinase 2 (MMP-2) and MMP-9 can enhance the development of diabetic retinopathy due to their pro-apoptotic actions^24^,^25^. Fig. 2D demonstrates that the increased CAS-3, MMP-2 and MMP-9 mRNA expression in *db/db* mice were substantially inhibited by DHI treatment.

Taken together, the results in Figs 1 and 2 suggest that administration of DHI to *db/db* mice can correct the diabetes-induced retinal structural abnormalities and inhibit the development of diabetic retinopathy. The action might be attributed to the anti-apoptotic effects of DHI.

### DHI inhibits the development of diabetic nephropathy

To determine if DHI can also protect *db/db* mice against nephropathy, urine samples for a 24 h duration at the different time points of treatment were collected and determined excreted nitrogen, creatinine and proteins. Table 1 shows that DHI had a little effect on both urea nitrogen and urine creatinine levels. However, DHI substantially reduced excretion of proteins indicating DHI protects the animals against diabetes-induced kidney dysfunction.

**Figure 3:**
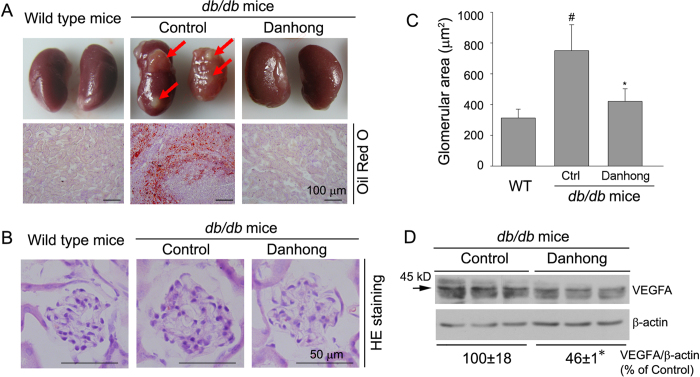
DHI inhibits lipid accumulation, and reduces total glomerular area and VEGFA expression in ***db/db*** mouse kidneys. **A**: the representative images of kidneys and Oil Red O staining of the kidney cross sections. Red arrows indicate the severe lipid accumulation in control *db/db* mouse kidneys; **B**: the representative images of glomerulus after HE staining; **C**: quantitative analysis of glomerular area of each groups. #, *: *vs.* wild type and control *db/db* groups, respectively, p < 0.05 (n = 5); **D**: expression of VEGFA protein in the cellular extract of kidneys was determined by Western blot. *: *vs.* control, p < 0.05 (n = 3).

At the end of study, kidneys were collected and checked their external appearance. Although most kidneys in control *db/db* mice appeared similar to wild type mice, the kidneys from 3 control *db/db* mice (total 10 mice) demonstrated abnormalities with un-matched size and severe lipid accumulation (top panel, Fig. 3A) which was confirmed by Oil Red O staining (bottom panel, Fig. 3A). However, none of such kind of kidney was observed in *db/db* mice receiving DHI treatment (Fig. 3A).

The diabetic nephropathy is initially characterized by thickening glomerular and tubular basal membrane with progressive mesangial expansion which ultimately leads to glomerulosclerosis and loss of renal functions. To determine the effect of DHI on kidney structure, in particular the glomeruli, kidney cross sections were HE stained and quantified glomerular area. Compared to wild type mice, the glomerular area was substantially increased in control *db/db* mice. However, DHI treatment inhibited the glomerular hypertrophy in *db/db* mice (Fig. 3B,C). High expression of VEGFA can make a contribution to the development of diabetic nephropathy and other glomerular diseases^16^. Compared to control *db/db* mice, DHI decreased VEGFA protein levels in kidneys (Fig. 3D).

**Figure 4:**
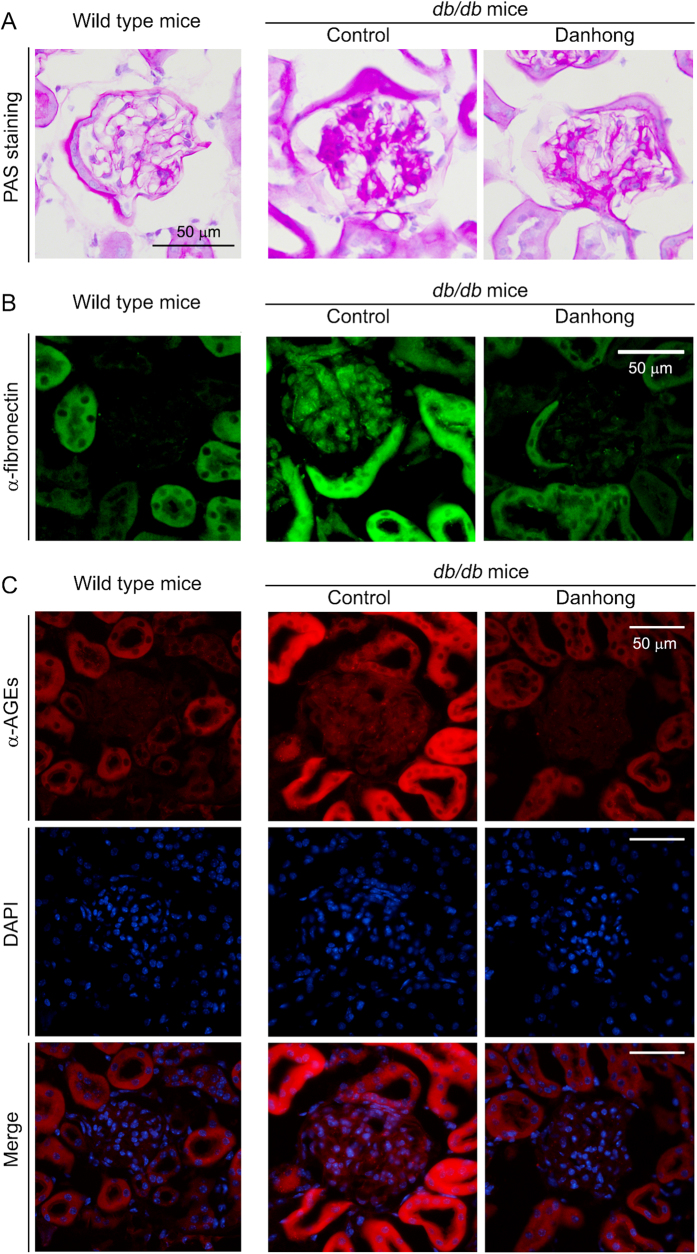
DHI inhibits the glomerular mesangial expansion, expression of fibronectin and accumulation of AGEs in ***db/db*** mice. **A**: the representative images of kidney cross sections after PAS staining; **B**, **C**: expression of fibronectin and accumulation of AGEs in glomeruli were determined by immunofluorescent staining with anti-fibronectin and AGEs antibodies, respectively.

The mesangial expansion in diabetic nephropathy is caused by mesangial cell proliferation, accumulation of carbohydrate macromolecules, such as glycogen, glycolipids and glycoproteins including extracellular matrix (ECM) proteins^26^. Compared to wild type mice, PAS staining demonstrates severe accumulation of macromolecules in glomeruli and increased mesangial expansion in control *db/db* mice. Treatment of the mice with DHI reduced these changes (Fig. 4A).

Fibronectin is a high-molecular weight (~260 kDa) glycoprotein of ECM and binds ECM components, such as collagen, fibrin and heparan sulfate proteoglycans. Expression of fibronectin is often used as an index to evaluate the levels of matrix accumulation in the presence of elevated glucose levels^27^. Fig. 4B shows a little and moderate fibronectin levels in glomeruli and tubules in wild type mouse kidney, respectively. Diabetes induced a high expression of fibronectin in both glomeruli and tubules. Interestingly, treatment of *db/db* mice with DHI decreased fibronectin expression in both glomeruli and tubules with a greater effect on glomeruli.

Formation of AGEs is another risk factor contributing to the development of diabetic nephropathy. Compared to wild type mice, diabetes induced AGEs accumulation in the kidney, mainly in tubules suggesting the induction of fibronectin expression and AGEs accumulation by diabetes might be in tissue/cell type-dependent manner^28^. Correspondingly, DHI administration blocked AGEs accumulation in tubules of *db/db* mice (Fig. 4C). Therefore, the results in Table 1 and Fig. 4 suggest that DHI also inhibits the development of diabetic nephropathy.

### The mechanisms by which DHI inhibits the development of diabetic retinopathy and nephropathy

**Figure 5:**
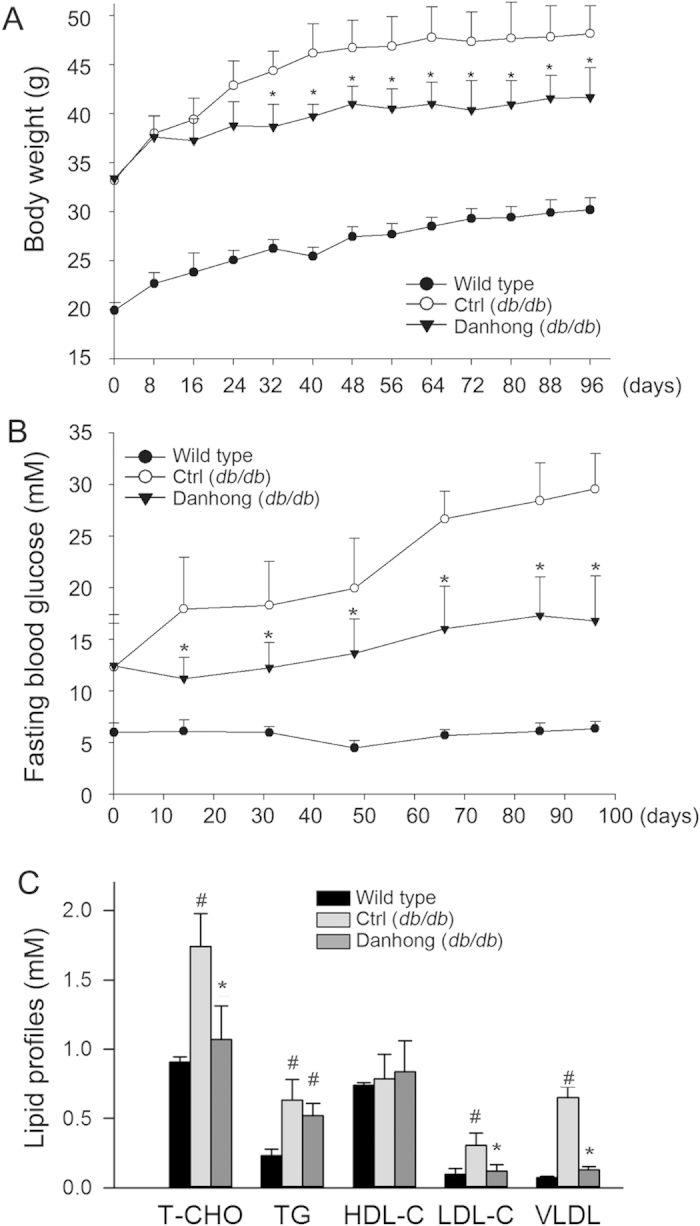
DHI inhibits diabetes-induced obesity, hyperglycemia and hypertriglyceridemia in **db/db**** mice. **A**: during the treatment, mouse body weight was routinely checked; **B**: blood was withdrawn for determination of fasting blood glucose levels at the indicated time points of treatment; **C**: mouse serum total cholesterol (T-CHO), triglyceride (TG), LDL-cholesterol (LDL-C), HDL-cholesterol (HDL-C) and VLDL-cholesterol (VLDL-C) were determined at the end of study. #, *: *vs.* wild type mice and control *db/db* mice respectively, p < 0.05 (n = 10).

During the treatment, we routinely checked mouse body weight. Fig. 5A shows that control *db/db* mice kept increasing body weight for the first 10 weeks. However, DHI significantly reduced body weight gain after one month treatment. At the end of study, body weight of control *db/db* mice was increased by ~43% (47.9 ± 0.6 g *vs.* 33.4 ± 0.4 g). However, DHI treatment reduced the increase to ~25% (41.6 ± 0.5 *vs.* 33.2 ± 0.4 g). In addition, we checked the food intake during the treatment and observed no significant difference between control *db/db* mice and mice receiving DHI treatment (based on the body weight).

Compared to low and constant blood glucose levels in wild type mice, the blood glucose levels in control *db/db* mice were increased with time (Fig. 5B) and more than two-fold increase was determined at the end of the study (29.6 ± 3.1 mM *vs.* 12.75 ± 1.1 mM). However, the increased glucose levels was substantially inhibited soon after DHI treatment (~two weeks). At the end, a moderate increased glucose levels (~30%, 16.79 ± 3.4 mM *vs.* 12.83 ± 1.0 mM) were observed in *db/db* mice receiving DHI treatment (Fig. 5B).

Serum total cholesterol (T-CHO) levels were much higher in control *db/db* mice than wild type mice owing to the higher TG-rich lipoprotein cholesterol levels, LDL- and VLDL-cholesterol, in particular VLDL-cholesterol (Fig. 5C). Treatment of *db/db* mice with DHI reduced total, LDL- and VLDL-cholesterol levels suggesting DHI can inhibit diabetes-induced hypertriglyceridemia. Therefore, the results in Fig. 5 suggest that inhibition of diabetic complications in *db/db* mice by DHI is associated with amelioration of serum glucose and cholesterol levels.

**Figure 6:**
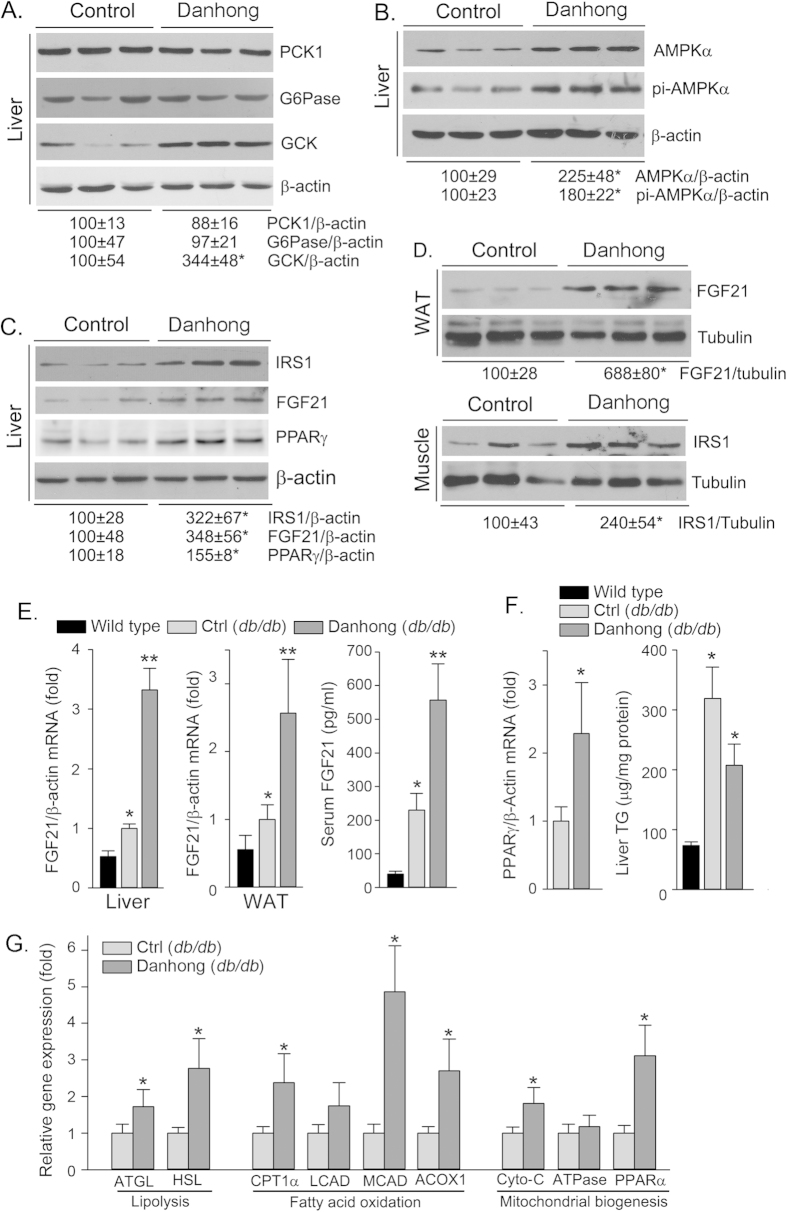
DHI activates expression of genes involved in glycolysis, insulin sensitivity and energy metabolism pathways in liver and peripheral tissues. After treatment, liver, white adipose tissue (WAT), muscle and blood samples were collected to prepare total cellular protein, total RNA, total lipid and serum, respectively. Expression of PCK1, G6Pase and GCK (**A**); AMPKα and pi-AMPKα (**B**); IRS1, FGF21 and PPARγ (**C**, **D**) protein was determined by Western blot, respectively. *: *vs.* control *db/db* mice, p < 0.05 (n = 3); Expression of FGF21 [E, *: *vs.* wild type mice; **: *vs*. control *db/db* mice, p < 0.05 (n = 3)], PPARγ [F, *: *vs.* control *db/db* mice, p < 0.05 (n = 3)], ATGL, HSL, CPT1α, LCAD, MCAD, ACOX1, Cyto-C, ATPase and PPARα [G, *: *vs.* control *db/db* mice, p < 0.05 (n = 3)] mRNA was determined by real time RT-PCR, respectively; Serum FGF21 levels [E, *: *vs.* wild type mice; **: *vs*. control *db/db* mice, p < 0.05 (n = 5)] and liver TG content [F, *: *vs.* wild type mice, p < 0.05 (n = 5)] were determined using assay kits, respectively.

Hyperglycemia is one of the major causes of diabetes. The intensive control of blood glucose can effectively reduce the risk of microvascular complications in type 2 diabetes patients^29^. To determine the mechanisms by which DHI reduces blood glucose (Fig. 5B), expression of several important molecules involved in glucose homeostasis and insulin sensitivity pathways in *db/db* mouse liver and peripheral tissues was assessed. DHI had little effect on both PCK1 and G6Pase expression (the 1^st^ and 2^nd^ up panels, Fig. 6A) indicating that DHI does not influence gluconeogenesis. However, DHI substantially induced GCK expression in the liver (the 3^rd^ up panel of Fig. 6A) which suggests activation of glycolysis. The induction of both AMPKα and pi-AMPKα expression (Fig. 6B) further suggests that glycolysis and fatty acid oxidation in the liver are activated by DHI. Moreover, DHI induces expression of IRS1, FGF21 and peroxisome proliferator-activated receptor γ (PPARγ, a ligand-activated transcription factor which increases insulin sensitivity^30^) in the liver (Fig. 6C). In addition, DHI induced FGF21 and IRS1 expression in white adipose tissue (WAT) and muscle in *db/db* mice, respectively (Fig. 6D). At the transcriptional level, we determined that induction of FGF21 and PPARγ protein expression in the tissues by DHI was associated with increased mRNA expression (left and middle panels of Fig. 6E, left panel of Fig. 6F). The induction of FGF21 expression also resulted in increased circulating FGF21 levels (right panel of Fig. 6E). Expression of PPARγ in the liver of diabetic mice may enhance the development of hepatic steatosis. Compared to wild type mice, liver TG levels were substantially increased in *db/db* mice. However, DHI did not increase liver TG levels indicating that DHI influences multiple molecules involved in hepatic lipid homeostasis and activation of PPARγ does not play a dominant role in liver TG levels.

Reduced body weight gain may contribute to improvement of diabetic phenotypes in *db/db* mice. To further understand the mechanisms by which DHI inhibits diabetic complications, we investigated the effect of DHI on energy metabolism by determining expression of genes involved in pathways of lipolysis, fatty acid oxidation and mitochondrial biogenesis. Both adipose triglyceride lipase (ATGL) and hormone-sensitive lipase (HSL) catalyze hydrolysis of TG into diacylglycerol and fatty acid, the first and rate-limiting step in TG hydrolysis although HSL can act on hydrolysis of diacylglycerol^31^,^32^. We determined that expression of ATGL and HSL was activated by DHI (left panel of Fig. 6G). Meanwhile, expression of some genes involved in fatty acid oxidation, such as peroxisomal acyl-CoA oxidase 1 (ACOX1), medium-chain acyl-CoA dehydrogenase (MCAD) and carnitine palmitoyltransferase 1A (CPT1α)^33^,^34^, in the liver was also induced by DHI (middle panel of Fig. 6G). Expression of cytochrome-C (Cyto-C) and PPARα indicates mitochondrial biogenesis (PPARα also controls fatty acid oxidation). The results in the left panel of Fig. 6G demonstrate that DHI induced expression of Cyto-C and PPARα.

Taken together, the results in Fig. 6 clearly demonstrate that DHI can activate signaling pathways for glycolysis, insulin sensitivity and energy metabolism thereby improving glucose metabolism as well as diabetic phenotypes.

## Discussion

Both diabetic retinopathy and nephropathy are frequently observed in the patients with a long history of diabetes. The diabetic retinopathy can eventually lead to blindness while the diabetic nephropathy is the leading cause of chronic renal disease and a major cause of cardiovascular mortality in the patients. In this study, we demonstrate that administration of DHI to *db/db* mice inhibited both diabetic retinopathy and nephropathy. The inhibitory effect is mainly attributed to the reduction of hyperglycemia. Mechanistically, DHI increased expression of GCK, AMPKα and pi-AMPKα in the liver indicating the glycolysis is enhanced. Meanwhile, DHI induced expression of IRS1, FGF21 and PPARγ in the liver or/and peripheral tissues which can increase insulin sensitivity. Induction of genes involved lipolysis, fatty acid oxidation and mitochondrial biogenesis suggests that DHI may enhance energy metabolism. Moreover, DHI inhibited CAS-3, MMP-2 and MMP-9 expression and formation of acellular capillaries in retinas that can prevent diabetes-induced apoptosis and protect retinas against diabetes-induced damage. Inhibition of VEGFA and fibronectin expression in the kidney by DHI can reduce mesangial expansion and accumulation of AGEs or other macromolecules, and ameliorate diabetes-induced proteinuria.

Hyperglycemia stimulates biosynthesis of ECM in mesangial cells which can cause glomerular dysfunction^35^. Meanwhile the hyperglycemia promotes CAS-3 expression which may enhance apoptosis and alterations of retinal structure^23^. High glucose levels also increase production of cellular reactive oxygen species (ROS), which can directly cause cell and tissue injury by apoptosis and oxidative stress in hyperglycemia^36^. In contrast, reducing oxidative stress demonstrates protective effects against diabetic complications^37^. DHI reduces oxidative stress by activating superoxide dismutase (SOD) and reducing production of malondialdehyde (MDA) in both myocardial ischemia/reperfusion (MI/R) and acute lung injury (ALI) mouse models^38^,^39^. Hydroxysafflor yellow A, salvianolic acid A/B and danshensu are the main active chemical constituents in DHI. They can reduce MDA production and increase SOD activity both *in vivo* and *in vitro*, respectively^38^,^40^. In addition, salvianolic acid A has been reported to inhibit oxidative stress-induced retinal pigment epithelial (RPE) cells apoptosis^41^, and decrease blood glucose levels by improving glucose metabolism in streptozotocin (STZ)-induced type 2 diabetic rats^42^. In this study, we demonstrate that DHI substantially decreased blood glucose levels implying the inhibition of diabetic retinopathy and nephropathy by DHI might be partly dependent on the control of glucose levels. AGEs accumulation is one of the major pathways involved in the development of diabetic complications. Recently, hydroxysafflor yellow A and salvianolic acid A have been proved to inhibit the formation and accumulation of AGEs^42^,^43^. In the cultured mesangial cells, salvianolic acid B inhibits high glucose-induced mesangial cell proliferation and fibronectin secretion^44^,^45^. *In vivo*, salvianolic acid B significantly suppresses the glomerular hypertrophy, mesangial expansion and renal fibronectin levels in STZ-induced diabetic rats^46^. In consistent with the above reports with chemical components, our study shows that DHI markedly decreased renal AGEs and fibronectin levels and inhibited glomerular hypertrophy and mesangial expansion in diabetic *db/db* mice.

Glucose homeostasis in mammals is tightly controlled through regulation of glucose production/catabolism in the liver and glucose uptake/utilization in peripheral tissues. In type 2 diabetes, decreased hepatic insulin sensitivity leads to altered glucose metabolism and thereby hyperglycemia^47^. DHI has no effect on expression of G6Pase and PCK1, the critical enzymes in gluconeogenesis. However, it substantially increased GCK expression which may activate glycolysis pathway in the liver (Fig. 6A). IRS1 is a major substrate participating in the insulin action in liver and skeletal muscle to enhance insulin sensitivity^48^,^49^,^50^. In our study, we demonstrate that DHI induced IRS1 expression in both liver and skeletal muscle of *db/db* mice. Activity of AMPKα in the liver is inversely related to the development of diabetes^51^. DHI increased both AMPKα and pi-AMPKα suggesting DHI activates energy metabolism which also contributes to anti-diabetic properties of DHI. Increased FGF21 can be observed in obese diabetic states. However, lack of FGF21 expression impairs PPARγ signaling thereby decreasing insulin sensitivity and hepatic lipid oxidation and triglyceride clearance in mouse models^52^,^53^. In contrast, treatment of the animals with FGF21 reduces blood glucose and TG levels, and improves insulin resistance^8^,^54^. Similarly, we determined that DHI increased FGF21 expression in tissues and circulation which may also make contribution to improvement of glucose metabolism.

Compared to *db/db* mice in the control group, DHI substantially reduced body weight gain after about one month treatment (Fig. 5A). The decreased body weight gain can contribute to the improvement of diabetic phenotypes. Mice in the control *db/db* group received the same volume saline injection as DHI while the food intake between the control and DHI groups is not significantly different. Thus, the decreased body weight gain in DHI group is not caused by injection itself or less food intake by DHI injection. In addition, the reduced blood glucose levels by DHI were observed ahead of the decreased body weight gain (Fig. 5A,B) which suggests it might be possible that the improvement of glucose metabolism results in the body weight gain is slowed. Expression of the genes involved in pathways of lipolysis, fatty acid oxidation and mitochondrial biogenesis was also activated by DHI (Fig. 6G) which further demonstrates that DHI can enhance energy metabolism. Consistently, AMPKα and PPARα, two molecules regulating energy metabolism, were also activated by DHI (Fig. 6B,G).

Taken together, our study demonstrates that the anti-hyperglycemic effects of DHI is partially completed by up-regulating genes expression for glycolysis, lipid oxidation, insulin sensitivity and energy metabolism pathways. Therefore, treatment of diabetic *db/db* mice with DHI inhibits the development of diabetic retinopathy and nephropathy simultaneously. Our study implies a potential alternative medicine for treatment of diabetic patients.

## Methods

### Materials

DHI was kindly provided by Jinan Buchang Pharmaceutical Co. Ltd (Shandong, China) and prepared as described^21^. Rabbit anti-caspase-3 (CAS-3), fibronectin, G6Pase and FGF21 polyclonal antibodies were obtained from Santa Cruz Biotechnology (Dallas, Texas). Rabbit anti-VEGFA, PCK1, GCK, IRS1 and PPARγ polyclonal antibodies were purchased from Proteintech Group (Chicago, IL). Goat anti-AGEs, rabbit anti-AMPKα and phosphorylated AMPKα (pi-AMPKα) polyclonal antibodies were purchased from Novus Biologicals (Littleton, CO). Triglyceride (TG) assay kit was purchased from Wako Chemicals (Neuss, Germany). All other chemicals were purchased from Sigma-Aldrich (St. Louis, MO) except as indicated.

### Animals

The protocols for *in vivo* study with mice were approved by the Ethics Committee of Nankai University (Tianjin, China), and the methods for *in vivo* study were carried out in accordance with the approved guidelines. Both male type 2 diabetic (BKS.C g-m +/+ Lepr^db^/J, *db/db*) and C57BLKS/J wild type mice (6-week-old) were purchased from the Animal Center of Nanjing University (Nanjing, China). The animals were maintained at the Animal Center of Nankai University with free to access food and drinking water.

According to the previous reports, the safety of DHI had been evaluated and the calculated LD_50_ of DHI was 39.5 ml/kg for mice by i.v. injection^55^. Based on the dose used in clinic, the dose of DHI for mice was converted to be 3–6 ml/kg body weight^56^. Therefore, male *db/db* mice were randomly divided into two groups (10 mice/group) and daily i.p. injected with saline (5 ml/kg body weight, control group) and DHI (5 ml/kg body weight, Danhong group), respectively. Male C57BLKS/J wild type mice were i.p. injected with saline as a nondiabetic or normal control.

### Determination of blood glucose, serum cholesterol and FGF21 levels

Blood was withdrawn from mouse tail vein after overnight fasting at the different time points after treatment and determined glucose levels with an OneTouch glucometer and test strips (LifeScan, Milpitas, CA) according to the manufacture’s instruction. At the end of treatment, all the animals were anesthetized and euthanized in a CO_2_ chamber. Blood samples were collected and kept for 2 h at room temperature (RT). After centrifugation for 20 min at 2,000 g, the serum was transferred into a new test tube and kept at −20 °C. To determine cholesterol levels, serum samples (~150 μl/sample) were diluted with 1xPBS (1:1), and then loaded onto an automatic biochemical analyzer (Model 7020, Hitachi, Tokyo, Japan) to determine cholesterol levels. Serum samples were also used to determine FGF21 levels using a mouse FGF21 ELISA kit (Boster, Wuhan, China) according to the manufacture’s instruction.

### Preparation of retinal vasculature, evaluation of retinal capillary basement membrane and determination of CAS-3 expression

The retinal vasculature was prepared based on the described method^57^ with minor modifications. Briefly, after enucleation mouse eyes were fixed in fresh 4% paraformaldehyde/PBS overnight. The retinas were dissected from the eyeballs and incubated in water overnight with gentle shaking at RT followed by digestion in 3% trypsin/PBS (Invitrogen, Grand Island, NY) for 2-3 h at 37 °C. The tissue was then transferred into filtered water and the network of vessels was freed from adherent retinal tissue by gentle shaking and manipulation under a dissection microscope. The vessels were then mounted on a clean slide and air-dried completely. The vessels were then stained with periodic acid-Schiff (PAS), washed with water, and dehydrated and mounted (Permount mounting medium, Fisher Scientific, Pittsburgh, PA). The prepared retinal vessels were observed and photographed under a microscope. Acellular capillaries were defined as capillary sized vessel tubes with no nuclei along their length^58^, and randomly counted with 4–6 filed areas around the mid-retina. Data are presented as number of acellular capillaries per 10 mm^2^ of retina.

To evaluate the retinal thickness and structural alterations, eyeballs were fixed in fresh 4% paraformaldehyde/PBS for 12 h at 4 °C, and then cryo-protected in 30% sucrose/PBS overnight before quick frozen in OCT compound solution (Sakura Finetek, Inc., Torrance, CA). The 5 μm frozen cross sections were prepared by a standard procedure. The sections were stained with hematoxylin and eosin (HE) for evaluation of the retinal structure. Expression of CAS-3 was determined by immunofluorescent staining as follows: the sections on cover slides were incubated with rabbit anti-CAS-3 polyclonal antibody overnight at 4 °C. After removal of the primary antibody by washing with PBS, the slides were stained with rhodamine-conjugated goat anti-rabbit IgG for 2 h at RT. After washed with PBS, the slides were re-stained with DAPI solution for nuclei. All the slides were viewed and photographed with a fluorescence microscope.

### Determination of renal functions

At the indicated durations, the animals were housed in metabolic chambers (Nalgene) for 24 h to collect urine samples. The urinary protein concentrations were determined by BCA method. The content of nitrogen and creatinine in urine samples were determined with the assay kits purchased from BioSino Bio-technology and Science Inc. (Beijing, China) according to the manufacturer’s instruction.

### Determination of glomerular area, accumulation of carbohydrate macromolecules, expression of VEGFA, fibronectin and AGEs in the kidney

After treatment, kidney 5 μm cross sections were prepared and conducted PAS or HE staining, and the glomerular area was quantified. The sections were also used to determine expression of fibronectin and AGEs by immunofluorescent staining. Expression of VEGFA in the kidney was determined with total cellular protein by Western blot as described^59^.

### Oil Red O staining

To determine lipid content, kidney frozen sections were rinsed with 60% isopropanol and then stained with freshly prepared Oil Red O working solution (3 mg/ml in 60% isopropanol) for 45 min. The sections were then rinsed with 60% isopropanol and lightly stained for nuclei with alum hematoxylin solution. The sections were finally rinsed with distilled water, mounted in an aqueous mountant, viewed and photographed with a microscope.

### Determination of protein expression of the glucose metabolism related genes in tissues

A piece of liver, muscle and white adipose tissue (WAT) were collected and used to extract total cellular protein followed by determination of protein expression of glucose metabolism related molecules by Western blot^59^.

### Determination of mRNA expression by real time RT-PCR

After treatment, mice retinas, a piece of liver and WAT were collected and homogenized in Trizol reagent (Invitrogen, Carlsbad, CA) to extract total cellular RNA. The cDNA was synthesized with the first-stand cDNA synthesis Kit from Fermentas (Pittsburgh, PA). Expression of CAS-3, MMP-2, MMP-9, FGF21, PPARγ, ATGL, HSL, CPT1α, LCAD, MCAD, ACOX1, Cyto-C, ATPase and PPARα mRNA was determined by real time RT-PCR using a SYBR green PCR master mix from Bio-Rad (Los Angeles, CA) and the primers listed in Table 2 and normalized by β-actin mRNA in the corresponding samples.

### Determination of liver TG content

To quantify TG content, a piece of liver (~50 mg) was homogenized in ~1.1 ml 1xPBS. A portion of the homogenate (100 μl) was saved for determination of protein content which was used to normalize TG levels; 1 ml homogenate was then used to extract total lipids followed by TG quantitative analysis with an assay kit.

### Data analysis

All experiments were repeated at least three times, and the representative results are presented. Data were presented as mean ± standard errors and analyzed by a Student’s t-test using Prism (GraphPad Software). The differences were considered significant at *p *< 0.05 (n ≥ 5).

## Additional Information

**How to cite this article**: Liu, M. *et al.* Administration of Danhong Injection to diabetic *db/db* mice inhibits the development of diabetic retinopathy and nephropathy. *Sci. Rep.* **5**, 11219; doi: 10.1038/srep11219 (2015).

## Figures and Tables

**Table 1:** **Effect of DHI administration on urinary excretion of nitrogen, creatinine and proteins in** ***db/db*** **mice.**

Day	Parameters					
	**Urea nitrogen (mM)**		**Urine creatinine (**μ**M)**		**Urinary proteins (mg/mouse/24h)**	
	**Control**	**Danhong**	**Control**	**Danhong**	**Control**	**Danhong**
21	133 ± 11	143 ± 11	129 ± 16	139 ± 24	943 ± 22	331 ± 38^¶^
54	117 ± 15	127 ± 21	117 ± 18	116 ± 31	1108 ± 37	543 ± 39^¶^
78	110 ± 14	125 ± 2	88 ± 19	112 ± 11	975 ± 14	749 ± 33^¶^
96	113 ± 13	129 ± 2	83 ± 6	86 ± 1	1287 ± 36	781 ± 16^¶^

The diabetic *db/db* mice at the age of 6-week old received daily i.p. injection of saline and DHI for 14 weeks. During the treatment, mice were placed in metabolic chambers at the indicated time points of treatment for collection of urine samples for 24 h durations. Levels of nitrogen, creatinine and proteins excreted into urine were determined. ¶: *vs.* controls, p < 0.05 (n = 10).

**Table 2:** **Sequences of the primers for real time RT-PCR analysis.**

**Gene**	**Forward**	**Backward**
CAS-3	GACTTGCTCCCATGTATGGTC	ATCAAAGCGCAGTGTCCTG
MMP-2	TGGCAAGGTGTGGTGTGCGAC	TCGGGGCCATCAGAGCTCCAG
MMP-9	GGTGTGCCCTGGAACTCACACG	AGGGCACTGCAGGAGGTCGT
b-Actin	ATCTGGCACCACACCTTC	AGCCAGGTCCAGACGCA
FGF21	AGGCCTCAGGATCAAAGTGA	CGCAGTCCAGAAAGTCTCCT
PPARγ	ATGTCTCACAATGCCATCAGGTT	GCTCGCAGATCAGCAGACTCT
ATGL	GAGCCCCGGGGTGGAACAAGAT	AAAAGGTGGTGGGCAGGAGTAAGG
HSL	GCCGGTGACGCTGAAAGTGGT	CGCGCAGATGGGAGCAAGAGGT
CPT1α	GCCCATGTTGTACAGCTTCC	TTGGAAGTCTCCCTCCTTCA
LCAD	TCACCAACCGTGAAGCTCGA	CCAAAAAGAGGCTAATGCCATG
MCAD	AGCTGCTAGTGGAGCACCAAG	TCGCCATTTCTGCGAGC
ACOX1	GAAGATGAGGGAATTTGGCA	CCTGATTCAGCAAGGTAGGG
Cyto-C	CCAGTCTTATGCTTGCCTCC	GGACGTCTGTCTTCGAGTCC
ATPase	TCTCCATGCCTCTAACACTCG	CCAGGTCAACAGACGTGTCAG
PPARα	AGTTCGGGAACAAGACGTTG	CAGTGGGGAGAGAGGACAGA

CAS-3, caspase-3; MMP-2, matrix metallopeptidase 2; MMP-9, matrix metallopeptidase 9; FGF21, fibroblast growth factor 21; PPARγ, peroxisome proliferator-activated receptor γ; ATGL, adipose triglyceride lipase; HSL, hormone-sensitive lipase; CPT1α, carnitine palmitoyltransferase 1A; LCAD, long-chain acyl-CoA dehydrogenase; MCAD, medium-chain acyl-CoA dehydrogenase; ACOX1, peroxisomal acyl-CoA oxidase 1; Cyto-C, cytochrome-C; ATPase, ATP synthases; PPARα, peroxisome proliferator-activated receptor α.
